# Direct visualisation and measurement of lung microstructure reveal insights into extracellular matrix dysregulation in COPD

**DOI:** 10.1186/s12931-026-03798-w

**Published:** 2026-07-03

**Authors:** Alex Chia Wei Kong, Tom Havelock, Michael Bennett, Cosma Mirella Spalluto, Karl J. Staples, Ashley Heinson, Kristoffer Ostridge, Hannah Burke, Anna Freeman, Aishath Fazleen, Benjamin Welham, Alastair Watson, Aruna T. Bansal, Jannie Marie Bülow Sand, Sarah Rank Rønnow, Bastian Angermann, Lisa Oberg, Adam Platt, Doriana Cellura, Maria Humbert, Tom Wilkinson

**Affiliations:** 1 Southampton NIHR Respiratory Biomedical Research Centre, University Hospital Southampton, Southampton, UK; 2 Clinical and Experimental Sciences, Faculty of Medicine, University of Southampton, Southampton, UK; 3 Early Clinical Development, Respiratory and Immunology R&D BioPharmaceuticals, Astrazeneca, Gothenburg, Sweden; 4 School of Clinical Medicine, University of Cambridge, Cambridge, UK; 5 Cambridge University Hospitals NHS Foundation Trust, Cambridge, UK; 6 Acclarogen Ltd, St John’s Innovation Centre, CB4 0WS Cambridge, UK; 7 Nordic Bioscience, Herlev, Denmark; 8 Translational Science and Experimental Medicine, Research and Early development, Respiratory and Immunology R&D BioPharmaceuticals, Astrazeneca, Gothenburg, Sweden; 9 Translational Science and Experimental Medicine, Research and Early Development, Respiratory & Immunology, BioPharmaceuticals R&D, AstraZeneca, Cambridge, UK; 10 Faculty of Medicine, VPD Heart and Lung Research Institute, University of Cambridge, Cambridge, UK

**Keywords:** COPD, Probe-based confocal laser endomicroscopy (pCLE), Extracellular matrix (ECM), Biomarkers, Early COPD

## Abstract

**Background:**

Extracellular matrix (ECM) dysregulation is a key process in the pathology of COPD. However, an inability to characterise ECM remodelling in vivo has limited our understanding of its relationship with functional decline and disease mechanisms. We aimed to quantify in vivo ECM remodelling using probe-based confocal laser endomicroscopy (pCLE) and determine associations with physiological, radiological, histological, and serological markers of COPD.

**Methods:**

16 patients with COPD and 20 controls underwent pulmonary function testing, CT imaging, bronchoscopy, and pCLE. Alveolar morphometrics and elastin linearity scores (ELS) were quantified using a novel automated algorithm. Bronchial biopsies were analysed for elastin and collagen content. Serum biomarkers of elastin and collagen turnover were measured in a combined cohort of 54 COPD patients and 61 controls.

**Results:**

Compared with never-smoking controls, current smokers without airflow obstruction demonstrated larger alveolar dimensions including increased alveolar opening area (AOA) (46,282 ± 16,805 vs. 33,549 ± 2,595 μm², *p* = 0.003). Alveolar dimensions were further increased in COPD, with larger AOA (56,468 ± 11,079 vs. 46,282 ± 16,805 μm², *p* < 0.001) compared with all controls. COPD was also associated with greater elastin fibre disorganisation (ELS 54.9 ± 6.0 vs. 47.5 ± 10.7, *p* = 0.032). Across the cohort, ELS correlated with airflow obstruction and surrogate markers of small airway disease. Airway collagen content was increased in COPD and correlated with ELS (*r* = 0.665, *p* = 0.005). COPD was associated with higher circulating elastin and collagen degradation biomarkers, including ELP-3, C1M, C6M, and EL-CG (all *p* < 0.05), which correlated with pCLE morphometrics.

**Conclusion:**

Using an innovative lung imaging technique, we provide the first objective quantification of in vivo airway elastic fibre disorganisation and demonstrate quantifiable lung microstructural changes that may precede abnormalities detected by established techniques. These quantifiable signals relate to biomarkers of lung ECM turnover, offering a new platform for early disease detection and mechanistic understanding of COPD.

**Supplementary Information:**

The online version contains supplementary material available at https://doi.org/10.1186/s12931-026-03798-w.

## Introduction

COPD is the third leading cause of death worldwide. However, no true disease modifying therapies exist to date [1]. A lack of understanding of early disease mechanisms and methods to detect and track early changes has limited the development of new diagnostic and therapeutic strategies to improve outcomes [2].

The extracellular matrix (ECM), comprised predominately of elastin and collagen, is central to the uniquely distensible nature of the lung and carrying vital elastic properties whilst maintaining patent airways and hence airflow. Elastin forms extensively cross-linked fibres, essential for its recoil properties [3], and collagen provides tensile strength which protects the alveoli from mechanical rupture during respiration [4]. Disruption to the intricate ECM homeostasis seen in COPD can lead to extensive small airways fibrosis which often precedes clinical recognition of airflow obstruction [5–7]. The complex interplay between inflammation, ECM regulation and the onset and progression of airflow obstruction remains unclear, largely because of our inability to quantify and track ECM pathology. Better understanding could yield new therapeutic opportunities to alter the natural history of disease progression.

Recent histological studies have highlighted altered collagen composition in COPD [8, 9]. Furthermore, pulmonary elastin degradation has been regarded as a key precursor to COPD, however, evidence of altered total elastin expression in the COPD lung parenchyma remains inconsistent [10]. A fundamental limitation of existing studies is their reliance on ex vivo histological assessment, which fails to capture dynamic ECM architecture. Consequently, the functional relevance of ECM dysregulation particularly in early COPD and during pre-clinical smoking exposure remains poorly defined.

Matrix metalloproteinases (MMPs) are known players in ECM dysregulation in smoke induced COPD [11–15]. Newly identified blood ECM degradation products offer new opportunities for identifying and stratifying patients at risk of early disease and have been linked to both disease progression and exacerbations, particularly neo-epitope fragments of elastin and collagen [16, 17]. Their direct relationship to ECM derangement however has not been explored to date and so uncertainty remains how they should be used clinically or in drug development.

Probe-based confocal laser endomicroscopy (pCLE), performed during bronchoscopy, enables real time visualisation of airway walls and alveolar architecture within the pulmonary tract. However, existing pCLE studies in COPD have been largely descriptive, underscoring the need for objective, quantitative methods to characterise ECM dysregulation in vivo [18, 19]. Furthermore, the relationship between pCLE detected ECM changes and established physiological markers of COPD remains to be established [20, 21].

We sought to characterise lung ECM changes in COPD using pCLE and novel analysis tools we developed. We correlated changes with lung function measures, CT measures of airways disease and potential novel blood biomarkers. Our study provides an understanding around novel approaches for detecting and monitoring early pathological changes in COPD and potential future therapeutic targets.

## Methods

### Study design and population

This study was conducted at The University Hospital Southampton NHS Foundation Trust, UK. The pCLE cohort contained 16 stable mild-to-moderate COPD patients (GOLD: post-bronchodilator FEV1/FVC < 0.70, FEV1 > 50% predicted, 5 ex-smokers and 11 current smokers) and 20 controls without airway obstruction, 10 never-smokers and 10 current smokers (Table 1) [22]. Participants underwent blood sampling, pulmonary function testing, high-resolution CT imaging, and bronchoscopy. Ethical approval was granted by the National Research Ethics Service South Central–Hampshire A (09/H0502/91).

**Table 1:** Demographics of the pCLE cohort

	Controls					
	Control Never Smokers (NS)	Control Current Smokers (CS)	*P* value NS vs. CS		COPD	*P* value All Controls vs. COPD (CS vs. COPD)
Subjects	10	10	-		16	-
Age, years	47 (7)	52 (7)	0.1558		62 (5)	**0.0003 (0.0293)**
Sex (M/F), n (%Males)	7/3 (70%)	4/6 (40%)	0.1775		11/5	0.4004 (0.1489)
Smoking pack year	0 (1)	33 (12)	0.0057		42 (17)	**0.0041** (> 0.9999)
FEV1% of predicted	107.8 (13.4)	115.1 (15.9)	> 0.9999		86.6 (13.9)	**0.0028 (0.0314)**
FEV1/FVC (%)	81.9 (3.7)	77.8 (3.6)	> 0.9999		63.2 (7.1)	**< 0.0001 (0.0139)**
MEF25-75%	98.6 (19.5)	61.2 (19.4)	0.3636		39.1 (12.6)	**< 0.0001 (0.0212)**
TLCO % of predicted	102 (14.0)	88 (14.2)	> 0.9999		84 (12.9)	0.4656 (> 0.9999)
E/I MLD median	0.75 (0.09)	0.78 (0.10)	> 0.9999		0.85 (0.04)	**< 0.001** (0.0841)
HRTCT %LAA-950	2.41 (2.01)	1.00 (2.11)	> 0.9999		7.19 (6.23)	**0.0026 (0.0047)**

Statistical testing performed using chi-squared for categorical variables (Sex: male or female) and Kruskal–Wallis with Dunn’s post hoc test comparing for continuous variables (all other variables). Significant values in bold (*p* < 0.05). COPD subjects comprised of 11 Current, 5 ex-smoker. Data are presented as median (standard deviation) unless otherwise indicated

Sex: male/female, n (% males); Smoking pack-years: cumulative tobacco exposure (packs/day × years); *FEV1% predicted* Forced expiratory volume in 1 s, expressed as % of predicted, *FEV1/FVC (%)* ratio of FEV1 to forced vital capacity, *MEF25–75% predicted* Mean forced expiratory flow between 25% and 75% of FVC, expressed as % predicted, *TLCO % predicted* Transfer factor for carbon monoxide, expressed as % predicted, *E/I MLD median* median ratio of expiratory to inspiratory mean lung density on CT, *HRCT %LAA-950* percentage of low-attenuation areas < − 950 HU on inspiratory high-resolution CT, reflecting emphysema

A sub-cohort of the pCLE cohort (the biopsy cohort) underwent elastin and collagen staining of bronchial biopsies (as below). The biopsy cohort comprised of 12 controls with preserved lung function (8 control never smokers and 4 control current smokers) and 9 COPD subjects (4 current smokers and 5 ex-smokers) (Table S1).

A subset of participants from the previously published Microbiology & Immunology of the Chronically Inflamed Airway (MICA) II [15] cohort included 39 ex-smoking subjects with mild-to-moderate COPD, 19 never-smoking controls and 22 ex-smoking controls (Table 2). These participants underwent blood sampling, pulmonary function testing, high-resolution CT imaging, and bronchoscopy.

**Table 2:** MICA II cohort

Study characteristics	Control never smoker	Control Ex smokers	COPD	*P* value
Subjects, n	19	22	39	-
Age	63.0 (9.0)	66.5 (7.9)	70.0 (7.9)	**0.0002**
Sex (M/F), n (%)	11/8 (58%)	12/22 (55%)	30/9 (77%)	0.1351
Smoking history (Never/Ex/Current)	19/0/0	0/22/0	0/39/0	**< 0.0001**
Smoking pack years	0 (2.0)	25 (12.5)	43 (24.6)	**< 0.0001**
FEV1 (%)	102 (14.6)	100.7 (14.0)	68 (16.2)	**< 0.0001**
FEV1/FVC (%)	79 (4.5)	77.7 (5.1)	55 (9.7)	**< 0.0001**
MMEF (%)	106 (29.5)	102.0 (25.4)	35 (16.1)	**< 0.0001**
E/I MLD median	0.80 (0.04)	0.80 (0.05)	0.87 (0.05)	**< 0.0001**
HRCT %LAA-950	4.8 (2.6)	5.4 (5.2)	10.5 (8.06)	**0.0006**

This cohort comprised of control volunteer never-smokers and COPD subjects from the previously published MICAII cohort. Statistical testing performed using chi-squared for categorical variables (Sex: male or female) and Mann-Whitney U test for continuous variables (all other variables). Data are presented as median (Standard deviation) unless otherwise indicated. Significant values in bold (*p* < 0.05)

Sex: male/female, n (% males); Smoking pack-years: cumulative tobacco exposure (packs/day × years); *FEV1% predicted* Forced expiratory volume in 1 s, expressed as % of predicted, *FEV1/FVC (%)* ratio of FEV1 to forced vital capacity, *MEF25–75% predicted* Mean forced expiratory flow between 25% and 75% of FVC, expressed as % predicted, *TLCO % predicted* Transfer factor for carbon monoxide, expressed as % predicted, *E/I MLD median* median ratio of expiratory to inspiratory mean lung density on CT, *HRCT %LAA-950* percentage of low-attenuation areas < − 950 HU on inspiratory high-resolution CT, reflecting emphysema

Blood biomarkers of ECM degradation were assessed in subjects from both the pCLE and MICA II cohorts combined, referred to as the neoepitope cohort. This combined cohort included 54 COPD subjects (43 ex-smokers and 12 current smokers) and 61 controls (29 never-smokers, 22 ex-smokers, and 10 current smokers) (Table S2).

### CT Thorax analysis

All High-resolution CT scans for subjects within the pCLE and MICA-II cohorts were performed using a Siemens Sensation 64 scanner and analysed with Apollo Software (VIDA Diagnostics). In total, 115 participants underwent CT imaging, of which 111 scans were included in the final analysis, four scans were excluded due to technical issues (two from the pCLE cohort and two from the MICA-II cohort). Emphysema was quantified by %LAA-950 (lung voxels with attenuation < − 950 HU) and air trapping by the expiratory-to-inspiratory ratio of mean lung density (E/I MLD), and airway thickening by airway wall area percentage (WA%) [23]. The imaging protocol consisted of; slice thickness 0.75 mm, slice separation 0.5 mm, tube voltage 120KV, effective mAs 90mAs, collimation 0.6 mm and a pitch of 1. The CT images were reconstructed to a slice thickness of 0.75 mm and a reconstruction increment of 0.5 mm on a sharp kernel reconstruction algorithm (B35f).

### Bronchoscopy and pCLE

Fibreoptic bronchoscopy with pCLE (Figure S1-S4) (Alveoflex, Mauna Kea Technologies) was performed imaging two lobes per subject. Figure S1 provides representative pCLE images from the various anatomical regions examined during bronchoscopy, orienting readers to the airway structures visualised by pickle and illustrating the identification of alveolar structures used for morphometric analysis.

Imaging of the distal lung was performed by advancing the pCLE probe into subsegmental bronchi of the right upper and right lower lobes. The probe was then advanced beyond the respiratory bronchioles, allowing direct visualisation of alveolar ducts. Alveolar openings were assessed at the level of the alveolar ducts, and measurements were derived from an average of 3–5 alveoli per subject across these. Video sequences were analysed for alveolar dimensions and elastic fibre structure, excluding poor-quality images [24]. Alveolar long-axis (LA) and short-axis (SA) diameters were measured (Figure S2), and alveolar opening area (AOA) calculated as π × (LA/2) × (SA/2). Five participants were excluded due to image quality. To reduce variability, a single rater analysed images for all patients. Furthermore, the rater was blinded to study group allocation to minimise risk of bias. Details about bronchial biopsy collection and analysis are provided in the supplement.

### pCLE Algorithm for elastin linearity scores

The algorithm for quantifying the angular distribution of elastic fibres was developed using Matlab (R2014a) (Figure S3 and S4. pCLE video frames acquired from the right main bronchus were processed to identify dominant fibre orientations, with linear features first enhanced and then segmented. The goal was to derive a single numerical value - the elastin linearity score (ELS), reflecting the dispersion of detected fibre angles. To achieve this, a Hough transform was applied to measure the orientation of individual fibres, and the standard deviation of these angles was calculated as the ELS. Further details of the ELS algorithm are provided in the online data supplement.

### Elastin and collagen neo-epitope biomarkers

Serum biomarkers of ECM turnover were quantified using validated ELISAs (Nordic Bioscience). PRO-C3, PRO-C4, and PRO-C6 measured collagen formation, C1M, C3M, C4M, and C6M measured MMP-mediated collagen degradation, and ELP-3, EL-CG, and EL-NE assessed elastin degradation [16]. Specifications for each biomarker are listed in Table S3.

### Movat’s pentachrome staining

Bronchial biopsies from the biopsy cohort (Table S1) were analysed to calculate the percentage area of elastin and collagen within the submucosa. Movat’s pentachrome staining quantified elastin and collagen, with measurements taken using ImageJ (See supplement for further details) [25]. RGB hue thresholding identified positive staining, excluding unrecognisable tissue and blood vessels.

### Statistical analyses

Statistical analyses were performed using SPSS V.21. Results are presented as mean (standard deviation), unless otherwise stated. Comparisons between groups were performed using Chi-Square, Mann–Whitney U, or Kruskal–Wallis tests as appropriate. Associations between pCLE findings, pulmonary function, CT parameters, biomarkers, and histological data were assessed using Spearman’s correlation.

To confirm the effect of demographics on biomarker neoepitope differences, additional analysis was performed using a logistic model which was fitted to regress Case/Control status on neoepitope, adjusting for smoking group, age, and sex. Smoking group was coded as a factor with three levels (1 = never; 2 = ex-; 3 = current smoker), however, smoking group was analysed as a quantitative (dose) parameter given only ten (9%) were current smokers, with values 1,2, and 3.

## Results

### pCLE alveolar diameters are larger in smoking and COPD and correlate with lung disease measures

We compared alveolar morphometrics using pCLE between 16 mild-to-moderate COPD subjects (11 current smokers and 5 ex-smokers) and 20 control subjects without airflow obstruction, 10 control current smokers (CS) and 10 control never-smokers (NS)) (Table 1). Overall, the cohorts were well matched for sex and other demographic characteristics, although COPD subjects were older than current smoker and never-smoker controls. There were no significant demographic differences between never smokers and current smokers, except expected differences in pack year history (Table 1).

**Fig. 1:**
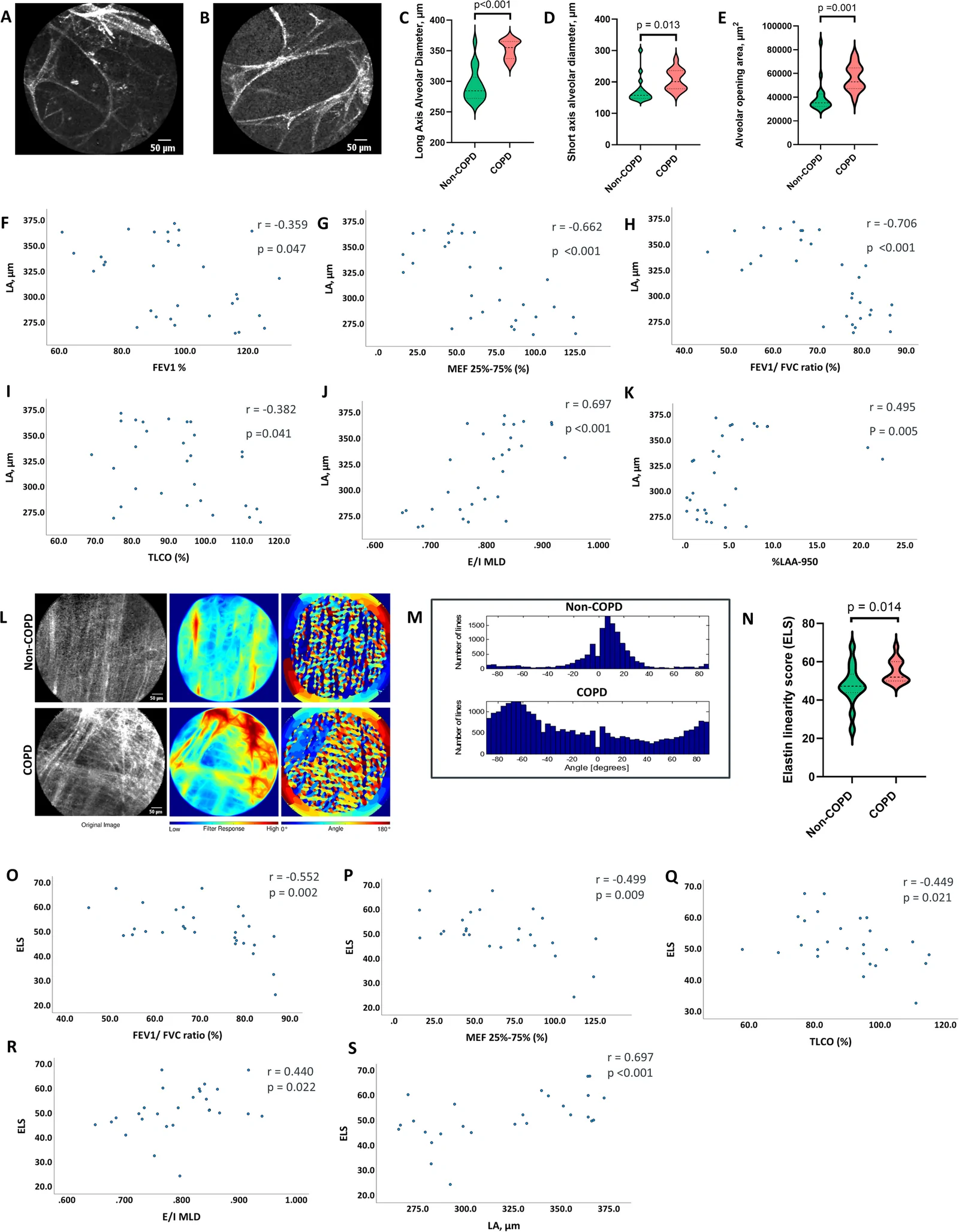
Probe-based confocal laser microscope defines dysregulated ECM in the lungs in COPD. Representative pCLE images demonstrating COPD (**B**) was associated with larger alveolar diameter compared to controls (**A**). **C**, **D**, **E** Violin plots comparing the long axis alveolar diameter, short axis alveolar diameter and alveolar opening area between controls and participants with COPD respectively in the pCLE cohort. Patients with COPD appear to have overall greater alveolar dimensions. Scatter plots of (**F**) Long axis alveolar diameter (LA) against FEV1%, (**G**) LA against MEF25%-75%, (**H**) LA against FEV1/FVC, (**I**) LA against TLCO%, (**J**) LA against E/I MLD and (**K**) LA against %LAA-950. N=31. **L** Elastic fibres (red/black) appear irregularly arranged in the main bronchus in a subject with COPD compared to a controls. **M** There is a clear broadening of the histogram confirming the distribution of elastic fibres is more disordered (greater ELS) in COPD. **N** Violin plot comparing ELS between controls with preserved lung function and participants with COPD. Scatter plots of (**O**) ELS against FEV1/FVC (**P**) ELS against MEF25%-75%, (**Q**) ELS against TLCO%, (**R**) ELS against E/I MLD and (**S**) ELS against LA, N=26

Current smokers had significantly larger short axis alveolar opening (SA) diameters (187.3 +/- 51.9 μm vs. 154.8+/-8.8 μm, *p* = 0.006) and alveolar opening area dimensions (33548.8 +/- 2594.7 µm^2^ vs. 46281.7+/-16805.2 µm^2^, *p* = 0.003) vs. never smokers (Table 3). Larger long axis alveolar opening (LA) 310.3 +/- 21.9 μm vs. 277.3 +/- 9.5 μm, *p* = 0.190) was also seen between current smokers and never smokers, although these differences did not reach significance. COPD subjects had larger alveolar opening area (56467.5 +/- 11078.9 µm^2^ vs. 46281.7 +/- 16805.2 µm^2^, *p* < 0.001), long axis alveolar opening diameter (352.3 +/- 15.5 μm vs. 310.3 +/- 21.9 μm, *p* < 0.001) and short axis alveolar opening diameter (207.2 +/- 33.5 μm vs. 187.3 +/- 51.9 μm, *p* < 0.001) vs. all controls (never smokers and current smokers combined) (*p* < 0.01) (Fig. 1C-E).

**Table 3:** Morphometric alveolar parameters and ELS measurements by study group

	Controls				
	Control Never smokers (*n* = 10)	Control Current Smokers (*n* = 10)	*P* value NS vs. CS	COPD (*n* = 16)	*P* Value COPD vs. Controls
Short axis alveolar diameter, µm	154.8 +/- 8.8	187.3 +/- 51.9	0.0056	207.2 +/- 33.5	< 0.0001
Long axis alveolar diameter, µm	277.3 +/- 9.5	310.3 +/- 21.9	0.1903	352.3 +/- 15.5	0.0007
Alveolar opening area, µm^2^	33548.8 +/-2594.7	46281.7 +/- 16805.2	0.0028	56467.5 +/- 11078.9	< 0.0001
ELS	43.8 +/- 10.3	54.0 +/- 8.9	0.1119	54.9 +/- 6.0	0.0141

Showing mean (standard deviation) unless otherwise stated. Control Never Smokers vs. Control current smokers compared with Mann Whitney Test in the pCLE cohort (Table 1). COPD vs. all controls compare using Mann Whitney test

*ELS* Elastin linearity score, *NS* Never smokers, *CS* Current smokers

Across the whole pCLE cohort, we explored the relationships of alveolar morphometrics with traditional markers of lung function, as well as surrogate CT and physiological measures of emphysema (high %LAA and low *T*_LCO_ % predicted, respectively) and surrogate CT and physiological measures of small airway disease (high E/I MLD and low MEF25-75%, respectively). Alveolar morphometrics long axis alveolar opening diameter, short axis alveolar opening diameter and alveolar opening area inversely correlated with FEV1% (*r*=-0.359, -0.464, -0.465, respectively, all *p* < 0.050), and FEV1/FVC (*r*=-0.706, -0.637 and − 0.721, respectively, all *p* < 0.001). Long axis alveolar opening diameter, short axis alveolar opening diameter and alveolar opening area correlated with surrogate CT marker of emphysema %LAA-950 (*r*=-0.495, 0.564 and 0.489, respectively, all *p* < 0.001). Long axis alveolar opening diameter negatively correlated with TLCO% (*r*=-0.382, *p* = 0.05). Furthermore, long axis alveolar opening diameter, short axis alveolar opening diameter and alveolar opening area significantly correlated with surrogate CT and physiological markers of small airways disease (high E/I MLD and low MEF25-75%), E/I MLD (*r* = 0.697, 0.612 and 0.677, respectively, all *p* < 0.001) and MEF25-75% *r*=-0.662, -0.645 and − 0.712, respectively, all *p* < 0.01), respectively.

### Elastin disorder is increased in COPD and correlates with CT and lung function measures of small airways disease

To understand for the first time how microstructural changes to the ECM relate to SAD in vivo, we next compared the level of elastin disorder seen during pCLE and caclulated elastin linearity scores using a novel algorythm developed within this study. Higher ELS values reflect greater elastin fibre disorganisation and loss of normal linear alignment. COPD subjects had greater elastin disorder (high ELS) vs. controls ((54.9(6.0)) vs. (47.5(10.7)), *p* = 0.032)) (Table 3; Fig. 1N). Across the whole pCLE cohort, ELS correlated with alveolar pCLE morphometrics seen; long axis alveolar opening diameter (*r* = 0.560, *p* = 0.003), short axis alveolar opening diameter (*r* = 0.522, *p* = 0.006) and alveolar opening area (*r* = 0.615, *p* = 0.001). Furthermore, ELS scores negatively correlated with FEV1/FVC (*r* =-0.599, *p* < 0.001), TLCO% (*r* =-0.458, *p* = 0.019), MEF25-75% (*r* = -0.614, *p* < 0.001) and RV/TLC (*r* = -0.395, *p* = 0.046) suggesting that in vivo elastic fibre disorganisation, which was visualised in the R main bronchus, is associated with physiological markers of small airways dysfunction. We also observed a significant direct correlation between ELS and CT measures of SAD: E/I MLD (*r* = 0.440, *p* = 0.022) and the airway wall area percentage (WA%), which is a CT marker of airway wall thickness, at the fourth-generation airways (*r* = 0.386, *p* = 0.046) and fifth-generation airways (*r* = 0.537, *p* = 0.004).

### COPD is associated with increased airway collagen deposition which correlates with disease severity measures

**Fig. 2:**
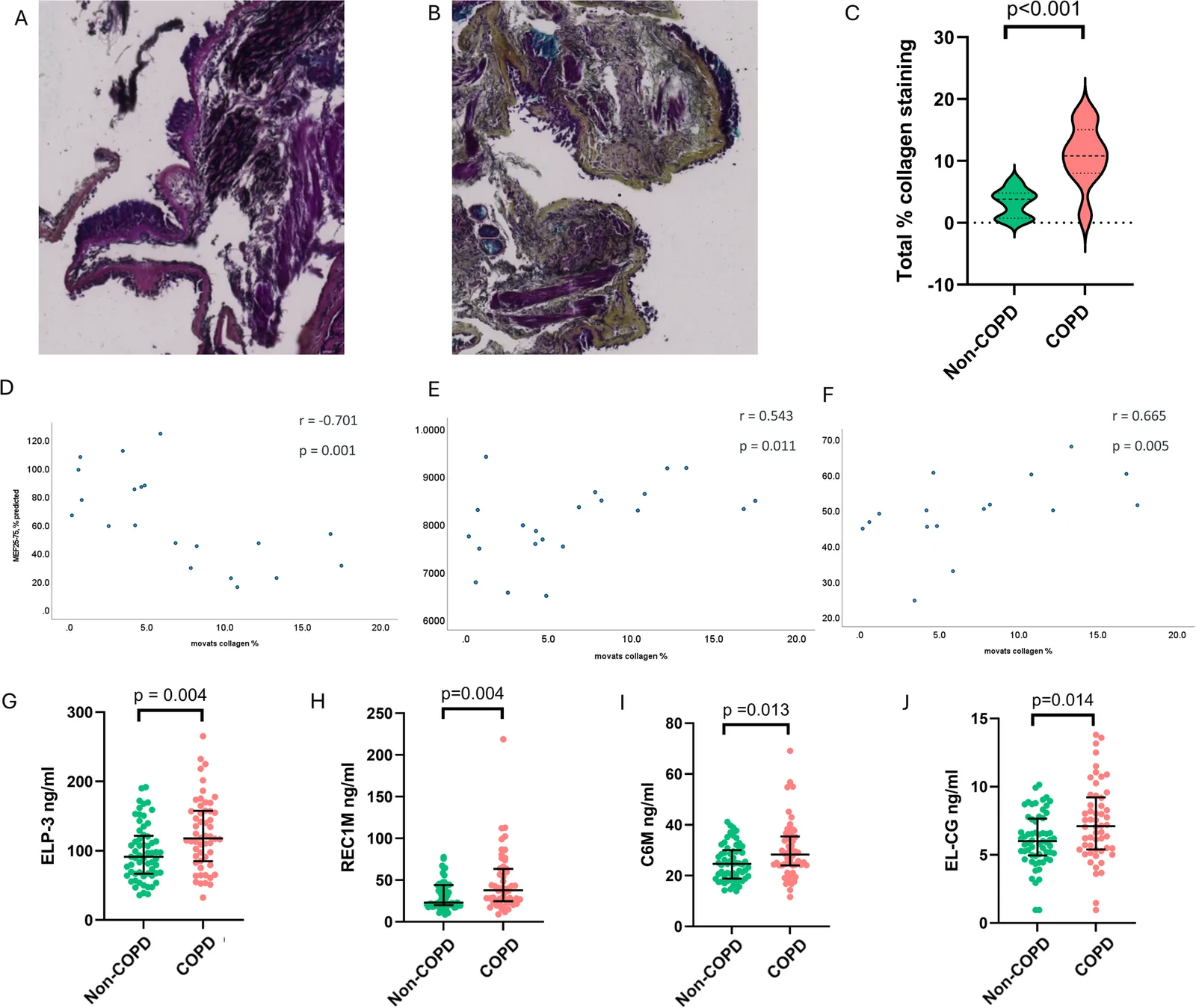
COPD is associated with increased deposition of collagen in the airways as well as elevated biomarkers of elastin and collagen turnover. Representative endobronchial biopsy tissue stained using Movat’s pentachrome in (**A**) controls and (**B**) COPD showing increased staining of collagen fibres (yellow) in COPD and no change in elastic fibres (black). **C** Violin plot comparing total percentage of collagen staining between control subjects with preserved lung function (n=12) and participants with COPD (n=9), respectively, from the biopsy cohort. N=21. Scatter plots of total percentage of collagen staining against FEV1/FVC (**D**) (n=20), MEF25%-75% (**E**) (n=21) and total ELS (**F**) (n=16). **G**, **H**, **I**, **J** Violin plots comparing neo-epitopes ELP-3, C1M, C6M and EL-CG between control subjects with preserved lung function and participants with COPD respectively, N=115; COPD=54, control=61

Next, we aimed to understand the existing morphometric data and in vivo changes seen in elastin disorder. To do this we quantified collagen and elastin staining from large airway biopsies from a subset of patients from the pCLE cohort, 12 controls with preserved lung function (8 control never smokers and 4 control current smokers) and 9 COPD subjects (4 current and 5 ex-smokers) (Demographics in table S1). Within this biopsy sub cohort, submucosal tissue collagen percentage was higher in the COPD group vs. controls (10.8%(5.0) vs. 3.8%(2.2), *p* < 0.001) (Fig. 2A-C). Across the whole biopsy sub cohort, collagen content correlated with ELS *r* = 0.665 (*p* = 0.005) as well as alveolar morphometrics; long axis alveolar opening diameter (*r* = 0.611, *p* < 0.01) and alveolar opening area (*r* = 0.507, *p* < 0.05). Furthermore, collagen content negatively correlated with FEV1/FVC (*r*=-0.629, *p* = 0.002) and FEV1% (-0.434, *p* < 0.05). Collagen content also correlated with surrogate CT measures of emphysema, and %LAA-950 (*r* = 0.594, p = < 0.05) (Table S4), and physiological and CT measures of small airways disease, MEF25%-75% (*r*=-0.701, *p* = 0.001) and E/I MLD (*r* = 0.543, *p* < 0.05), respectively (Fig. 2D-F). Contrasting to differences seen in collagen, there was no significant difference in the submucosal tissue elastin percentage between groups (Table S4).

### Serological neoepitope markers of ECM correlate with pCLE findings and disease measures in COPD

To determine whether aberrant ECM remodelling is detectable in the blood in COPD and to gain insights about potential novel biomarkers, we analysed blood neoepitopes which reflect enzyme-specific degradation of elastin and collagen in a cohort of 54 COPD patients (43 ex-smokers and 11 current smokers) and 61 non-COPD subjects (29 never smokers, 22 ex-smokers and 10 current smokers), the neoepitope cohort (Table S2; comprised of subjects from pCLE cohort and previously reported MICAII cohort combined). Within this cohort, significant differences were seen between COPD and controls in terms of age (median (standard deviation) of 67 [8] vs. 59 [11], respectively, *p* < 0.001), proportion of males (74.5% vs. 55.7%, respectively, *p* < 0.001) and smoking history, 42(22.7) vs. 10(17.2) pack years, respectively, and expected spirometry differences. There were no differences in blood biomarker levels between control never smokers and control ex-smokers. However, COPD was associated with higher levels of elastin and collagen degradation biomarkers vs. controls, including ELP-3 (mean(Standard deviation)), 117.9(72.7)ng/ml vs. 91.3(54.9)ng/ml, *p* = 0.004), C1M (37.7(38.9)ng/ml vs. 33.2(24.3)ng/ml, *p* = 0.004), C6M (28.3(11.3)ng/ml vs. 24.6(11.2)ng/ml, *p* = 0.013) and EL-CG (7.1(3.8)ng/ml vs. 6.01(2.7)ng/ml, *p* = 0.014) (Fig. 2G-J). There were no differences between groups for PRO-C3, PRO-C4, C3M, C4M and EL-NE. To confirm these neoepitope differences, a logistic regression model was fitted to adjust for smoking group, age, and sex. After doing this, C1M, C6M and EL-CG remained significantly different, *p* < 0.05 for all, Pro-C4 was also significantly different *p* = 0.0388. However, ELP-3 was no longer significantly (Table S5).

To investigate whether potential peripheral blood biomarkers reflect underlying ECM derangement and lung pathology captured in vivo, we correlated serum neoepitopes with pCLE measures of alveolar morphometry, ELS, lung function, and CT and physiological markers of small airways disease in the neoepitope cohort (Table S2 and Table S6). Circulating markers of elastin degradation, EL-CG and ELP-3, were associated with increased pCLE short axis alveolar opening diameter (*r* = 0.398 and *r* = 0.416, respectively, both *p* < 0.05) and inversely correlated with markers of airflow limitation, including FEV₁% (r-0.240 (*p* < 0.05) and *r*=-0.410 (*p* < 0.01), respectively), FEV₁/FVC (*r*=-0.268 and *r*=-0.321, respectively, both *p* < 0.01), and MEF25–75% (*r*=-0.309 (*p* < 0.01) and *r*=-0.239 (*p* < 0.05), respectively) (Table 4). EL-CG correlated with CT measure of small airways disease, E/I MLD *r* = 0.240, *p* < 0.05). EL-NE and C6M were positively correlated with, ELS *r* = 0.542 (*p* < 0.01) and *r* = 0.384 (*p* < 0.05), respectively. Collagen degradation marker C1M negatively correlated with lung function measures, FEV1%, FEV1/FVC and MEF25-75% (*r*=-0.314, *r*=-0.253 and r-=0.68, all *p* < 0.01). C4M correlated with alveolar morphometric markers short axis alveolar opening diameter and alveolar opening area (*r* = 0.463 and *r* = 0.394, respectively, both *p* < 0.05) and negatively correlated with FEV1% (*r*=-0.195, *p* < 0.05) and MEF25%-75% (*r*=-0.268, *p* < 0.05). C6M correlated with short axis alveolar opening diameter and alveolar opening area (*r* = 0.364 and *r* = 0.384, respectively, both *p* < 0.05) and negatively correlated with FEV1%, FEV1/FVC and MEF25-75% (*r*=-0.280, *r*=-0.220 and r-=0.261, all *p* < 0.05) (Table 4).

**Table 4:** Spearman’s correlation analysis between neoepitope biomarkers, lung function, CT measures and pCLE parameters

EL-CG	FEV1%	FEV1/FVC	MEF25%-75%	E/I MLD	SA	LA	ELS	AOA
	-0.240*	-0.268**	-0.309**	0.240*	0.398*	0.094	0.283	0.309
ELP-3	-0.410**	-0.321**	-0.239*	0.163	0.416*	0.166	0.271	0.359
EL-NE	-0.091	-0.060	0.021	0.048	0.049	0.123	0.542**	-0.030
C1M	-0.314**	-0.253**	-0.268**	0.113	0.318	0.073	0.135	0.227
PRO-C3	0.150	0.064	0.044	-0.183	0.443*	0.158	0.162	0.284
C3M	-0.190*	-0.108	-0.166	-0.003	0.424*	0.022	0.263	0.286
PRO-C4	-0.038	-0.063	-0.181	0.079	0.475*	0.230	0.211	0.398*
C4M	-0.195*	-0.147	-0.268**	0.012	0.463*	0.132	0.286	0.394*
C6M	-0.280**	-0.220*	-0.261**	0.126	0.364*	0.232	0.384*	0.335

Analyses performed on the neoepitope cohort (Table S2), 61 controls and 54 COPD subjects (115). Numbers in the boxes are the r value of statistically significant correlations *= *p* < 0.05, **= *p* < 0.01. Significant values in bold (*p* < 0.05)

*FEV1% predicted* Forced expiratory volume in 1 s, expressed as % of predicted, *FEV1/FVC (%)* ratio of FEV1 to forced vital capacity, *MEF25–75% predicted* Mean forced expiratory flow between 25% and 75% of FVC, expressed as % predicted, *E/I MLD* ratio of expiratory to inspiratory mean lung density on CT, *SA* Short-axis alveolar opening diameter, *LA* Long-axis alveolar opening diameter, *AOA* Alveolar opening area, *ELS* Elastin linearity score, *EL-CG* cathepsin G generated elastin fragment, ELP-3 proteinase 3 generated elastin fragments, *EL-NE* neutrophil elastase-generated elastin fragments, *C1M* matrix metalloproteinase-generated type I collagen fragments, *PRO-C3* type III collagen formation marker, *C3M* matrix metalloproteinase-generated type III collagen fragments, *PRO-C4* type IV collagen formation marker, *C4M* matrix metalloproteinase-generated type IV collagen fragments, *C6M* matrix metalloproteinase-generated type VI collagen fragments

## Discussion

This study integrates in vivo bioimaging with biomarker, physiological and CT measures of disease to validate a novel pCLE-based imaging technique for assessing alveolar dimensions and airway elastin remodelling in COPD. We demonstrate that microstructural alveolar changes and elastic fibre disruption correlate with markers of airflow limitation, small airways disease, and emphysema. Importantly, changes were detectable in smokers prior to the development of lung function abnormalities measured using conventional techniques. We confirm that ECM derangement of both elastin and collagen are hallmarks of COPD, supported by immunohistochemistry and serological neo-epitopes and offer, for the first time, a way of quantifying microarchitectural changes that may occur well in advance of current approaches to detect and track disease using CT and lung function measures.

Our findings that smokers with preserved lung function exhibit enlarged alveolar opening diameters highlights that ECM remodelling could occur before detectable declines in lung function in smoking. Here histological studies have previously established a link between enlarged alveolar airspaces and emphysema [20, 21, 26, 27]. Our findings suggest that alterations in ECM and pulmonary architecture may precede clinically measurable lung function decline and highlights the potential of pCLE as an in vivo tool for discerning early pre-clinical COPD ECM disruption and studying the onset of disease. Nevertheless, these findings should be interpreted with caution as the cross-sectional design and absence of ex-smoker controls limits our ability to determine whether these changes are attributable to ongoing smoking, are potentially reversible, or represent early extracellular matrix remodelling prior to the development of COPD.

Our work also shows that increased elastic fibre misalignment and matrix disorder in COPD subjects relates to markers of airway wall thickening, airflow obstruction and pCLE-derived alveolar metrics. To enable consistent and reproducible quantification of elastin linearity, ELS was measured in the right main bronchus. Whilst this reflects ECM remodelling in larger airways, its strong correlations with pCLE-derived alveolar morphometrics and physiological markers of small-airways disease suggest that these changes may also reflect distal airway pathology. However, this needs to be interpreted carefully as elastin fibre organisation across different airway regions likely have different spatial heterogeneity of airway remodelling. Importantly, a previous study in asthma demonstrated good concordance between in vivo pCLE-derived elastic fibre patterns and airway histology, supporting the validity of this approach for assessing ECM remodelling [28]. Furthermore, a study in COPD demonstrated a higher prevalence of a “loose” elastin fibre pattern on pCLE in COPD which was corroborated with histological findings on biopsy analysis [21]. However, within this present study, it was not possible to undertake a direct one-to-one spatial correlation between pCLE-derived ELS and histological elastin architecture. This represents a limitation of this study which needs further exploration in the future. Nevertheless, we show how this novel in vivo imaging tool allows the direct detection of microstructural changes of alveolar architecture, as well as elastin disorder within the large airways, this may have utility in advancing disease understanding and in the development and validation of new imaging biomarkers within a research setting. Notably, this tool could also add value in early-phase trials of disease-modifying therapies, where sensitive detection of microstructural changes may provide a more responsive endpoint of treatment.

We complement the pCLE findings by showing increased airway collagen staining in COPD vs. healthy controls, helping explain previous findings which link emphysema to collagen deposition and airspace size with lung collagen levels [29]. Experimental models show chronic cigarette smoke exposure reduces total lung collagen but increases collagen deposition around small airways, indicating a fibrotic repair process [30]. Collagen density is elevated in mild to moderate COPD but declines in severe disease, suggesting early fibrotic remodelling followed by ECM degradation [31]. Second harmonic generation microscopy reveals increased immature, disorganised collagen in COPD airways [32], aligning with our findings of accelerated collagen turnover, supported by elevated collagen neoepitopes.

It is important to consider that elastin is a highly stable protein, whereas collagen is more dynamically regulated, undergoing continuous synthesis and degradation throughout life. Elastin degradation has been shown to be accompanied by increased collagen deposition, reflecting a shift in ECM remodelling rather than a simple loss of matrix components [33]. These differences highlight that ELS may be more closely related to alterations in elastin architecture—particularly fibre disorganisation—rather than absolute elastin quantity. The relationships between ELS, elastin, and collagen should also be interpreted within the broader context of coordinated ECM remodelling, as the ECM represents a highly complex and dynamic biological system with intricate cellular and molecular interactions and disruption to this likely underpins COPD pathophysiology.

Our data highlight the potential of novel ECM-derived protein biomarkers which offer valuable insights into tissue remodelling, disease activity, and could hold potential for monitoring treatment responses in COPD [5]. Our finding of elastin degradation biomarkers, which are mediated by PR3 and CG, being elevated in COPD aligns with their known role in neutrophilic inflammation and mucus hypersecretion [34, 35]. Collagen type III (C3M), type IV (C4M, C4Ma3), and type VI (C6M) have previously been found to be elevated in stable COPD [16]. We add context to these studies through finding significant correlations of elastin and collagen degradation biomarkers (Type I, III, IV, VI) with conventional airflow limitation measures, physiological measures of small airways disease and in vivo imaging, showing increased alveolar airspace and loss of elastin homeostasis. Our results help delineate the importance of ECM remodelling in COPD pathogenesis and highlight the potential utility of ECM neoepitopes to guide diagnosis and predict disease activity, clinical prognosis and as potential intermediate endpoints for clinical trials of therapeutic strategies aiming to slow disease progression.

We describe ECM dysregulation using novel in vivo imaging techniques and biomarkers which significantly correlate with CT measures of disease activity. However, the power of our analyses may have been limited by the relatively small sample sizes. We acknowledge that sampling error is a challenge, as pCLE measurements were obtained from selected airway regions and may not fully capture the heterogeneity of airway pathology. Nevertheless, the observed correlations with physiological indices, CT metrics, and histological staining support the relevance of these findings. Use of multiple cohorts and comparison of subjects with differing ages, comorbidities and smoking histories also adds complexity to interpretation. It is not possible to fully rule out the effect of these potential confounders, particularly due to the likely influence of systemic factors on serological neoepitope markers. Smoking status may influence extracellular matrix (ECM) structure and remodelling. The differing smoking statuses therefore may have impacted the results within this study. To investigate this, we performed a logistic regression adjusting for age, sex and smoking status. Notably the neoepitopes remained different between groups other than ELP-3 where the association was dampened, suggesting that these demographics may play a role in the differences seen for this molecule, smoking is well known to drive MMP expression in COPD [11–15]. Notably, we see dimensional microstructural differences in COPD vs. controls, this is not reflected in differences in elastin disorder which raises the question about whether smoking status has influenced the correlations of ELS with disease metrics within this study, which could reflect elastin disorganisation due to early smoking-related changes. Further studies matched for smoking history in more advanced disease are indicated to more fully account for smoking history and to determine whether ELS progressively worsens with increasing COPD severity. Furthermore, confirmation in larger, age-matched cohorts enriched for small-airways disease is warranted, alongside work examining less abundant ECM proteins, such as fibronectin and glycoproteins, which are likely to be integral to COPD pathogenesis. Although pCLE shows promise for visualising ECM remodelling in COPD, future studies should also assess the reproducibility of this technique, including inter-operator and inter-rater variability, longitudinal repeatability, and automated ELS quantification.

## Conclusion

Novel approaches for identifying and treating early origins of COPD disease are vital if disease modifying interventions are to be developed to meet the global unmet clinical need of this disease. We demonstrate the potential of a novel pCLE approach by objectively quantifying alveolar, ECM and airway microscopic changes for the first time. Correlating these novel indices with potential biomarkers of disease activity and indices of small airways disease and emphysema in COPD offer new opportunities for earlier diagnosis and disease activity monitoring, underscoring the central role of ECM remodelling as a novel target for new therapies in COPD.

## Supplementary Information

Supplementary Material 1.

## Data Availability

All data supporting the findings of this study are available within the paper and its Supplementary Information. Further queries will be responded to by email request.
