# Supplementary material for: Direct visualisation and measurement of lung microstructure reveal insights into extracellular matrix dysregulation in COPD
Source: Respir Res. 2026 Jul 3;27:367. doi: 10.1186/s12931-026-03798-w (PMC13599106; doi:10.1186/s12931-026-03798-w)
Supplement: Supplementary file 1 [file 12931_2026_3798_MOESM1_ESM.docx]

**Online Supplement:**

**Direct Visualisation and Measurement of Lung Microstructure Reveal Insights into Extracellular Matrix Dysregulation in COPD**

## Supplementary Methods

## pCLE algorithm description for ELS

The pCLE video sequences of the right main bronchus were manually inspected, and frames that clearly contained only noise (i.e. with no airway wall contact) were excluded. The algorithm was then applied to one continuous sequence of video frames, specifically the longest usable sequence identified. No other criteria were applied to frame selection.

A novel image processing methodology was developed by our team to quantify the angular distribution of elastic fibres (Figure S4). Custom software was implemented in Matlab (R2014a, The Mathworks, Inc., Natick, Massachusetts, United States) to apply this algorithm to the pCLE video frames obtained from the Cellvizio platform.

Detection of the elastic fibres in pCLE images is complicated because the fibres themselves do not form the boundaries between different image regions, they have a discrete thickness which is greater than one pixel and the signal-to-noise ratio of the images is relatively low. A method based on a bank of Gabor filters which function to determine the dominant direction at each pixel in the image was developed. This direction was in turn used to orientate an asymmetrical Gaussian filter, giving an anisotropic filtering system that would enhance linear features in the image and suppress the noise sufficiently to enable further analysis to determine the mathematical properties of the linear features in the image. The process is described in more detail below. All algorithms were implemented in MATLAB (MathWorks, Natick, MA).

### Pre-processing

Video data captured during the pCLE process was exported as a series of PNG files which were used for all subsequent analysis. Owing to the nature of the pCLE process the exported sequence of frames would contain a mixture of frames containing valid images of elastic fibres and those containing just noise or otherwise no useful information. An automated process to detect valid frames, and the regions within those frames that contained valid information, was developed based on a convolutional neural network approach. Manual labelling was performed over a subset of the data identifying valid regions of images and these labels were used to train the neural network accordingly. The trained network was then used to perform semantic segmentation to yield masks identifying valid regions for further processing in each image.

These masks were used to exclude all extraneous detail from the images which were then processed by applying a 5 by 5 median filter followed by a 3 by 3 averaging filter.

### Anisotropic filtering

Gabor filters are selective in terms of both the scale and orientation of the features to be detected. For the work described here it was assumed that the scale was constant and therefore a filter bank was constructed consisting of 16 Gabor filters with orientation ranging from 0 to $\pi$ radians.

Gabor filters were implemented according to:

$$g_{n}\left( x,y \right)\text{=}\frac{1}{2\pi\sigma_{x}\sigma_{y}}exp\left( \text{-}\frac{1}{2}\left[ \frac{x^{'2}}{\sigma_{x}^{2}}\text{+}\frac{y^{'2}}{\sigma_{y}^{2}} \right] \right)exp\left( \frac{j2\pi}{\lambda x^{'}} \right)$$

$$x^{'}\text{=}xcos\theta\text{+}ysin\theta$$

$$y^{'}\text{=-}xsin\theta\text{+}ycos\theta$$

$$\theta\text{=}\left( \frac{n}{16} \right)\pi$$

$$n\text{=}\left[ 0\ldots15 \right]$$

In this case, $\lambda$corresponds to the spatial wavelength of the harmonic function and effectively sets the scale selectivity of the filter. For the scales being considered in this context, a value for $\lambda$of 25 pixels was found to be effective.

The values for $\sigma_{x}$ and $\sigma_{y}$ were chosen to be equal giving a symmetrical Gaussian windowing function for the harmonic component of the filter. In practice a value of 10 pixels was found to give good results.

The resulting filter bank was used to determine the dominant direction at each location in the image according to:

$$I_{dir}\text{=}arg\max_{n}\left( \left| g_{n}\text{*}f \right| \right)$$

Where $f$represents the pre-processed image.

An example of the results obtained for $I_{dir}$ may be seen in Figure S4 (top row).

The final output of this stage of the process was obtained by filtering the input image with anisotropic Gaussian filters with an angle of rotation set to the value of $I_{dir}$ for each pixel. These filters were constructed according to:

$$h_{n}\left( x,y \right)\text{=}\frac{1}{2\pi\sigma_{x}\sigma_{y}}exp\left( \text{-}\frac{1}{2}\left[ \frac{x^{'2}}{\sigma_{x}^{2}}\text{+}\frac{y^{'2}}{\sigma_{y}^{2}} \right] \right)$$

$$x^{'}\text{=}xcos\theta\text{+}ysin\theta$$

$$y^{'}\text{=-}xsin\theta\text{+}ycos\theta$$

$$\theta\text{=}\left( \frac{I_{dir}\left( x,y \right)}{16} \right)\pi$$

$$n\text{=}\left[ 0\ldots15 \right]$$

These Gaussian filters were designed to be asymmetrical with $\sigma_{y}\text{=}0.25\sigma_{x}$. It was found that a value for $\sigma_{x}$ of 3 pixels gave good results as shown in Figure S3 (lower row).

The Gabor and anisotropic Gaussian filters were implemented using modified versions of the gabor_fn function by Dr. Deepak Kumar Rout obtained from the MATLAB Central File Exchange (1).

### Extraction of linear features

Following enhancement of the desired linear features by the above process, the next step was to automatically detect and describe the linear features.

This was achieved by first applying Frangi filtering to isolate linear features. Frangi filtering was implemented using the fibermetric function provided by the MATLAB Image Processing Toolbox, with just one parameter corresponding to the linear width which was set to 20 pixels.

Thresholding and morphological operators were applied to remove all irrelevant features after which the result was converted to a binary image and skeletonized to yield single pixel width lines corresponding to the linear features in the original image.

The Standard Hough Transform, implemented using functions from the MATLAB Image Processing Toolbox, was then used to detect the properties of these lines. Of these properties, of most interest was the location of the start and end points which were used to display the lines overlayed onto the pCLE image data, and the orientation $\theta$.

The standard deviation of the values of $\theta$ for all the lines in each frame was computed. After all the frames for one subject were analysed, the mean of the standard deviation values was computing giving a single number representative of the degree of angular distribution of the detected elastin fibres (the Elastin Linearity Score, or ELS).

## Bronchial biopsies

Bronchial biopsies were collected during bronchoscopy and analysed. Two airway mucosal biopsies were collected for immunohistochemistry and placed in 5 mL of 10% neutral buffered formalin (NBF) on a shaking platform for 6 hours to overnight at room temperature. After formalin fixation, the bronchial biopsy samples were dehydrated through a series of alcohol solutions with increasing concentrations (70% isopropyl alcohol (IMS), 100% IMS, 100% IMS, Xylene, Xylene; approximately 2 minutes in each with agitation). Subsequently, the tissue samples were infiltrated with molten paraffin wax. The samples were then transferred to moulds, and molten paraffin was added to embed the tissue. Once the paraffin solidified, the tissue became embedded in a paraffin block for subsequent analysis using Movat’s pentachrome stain.

## Movat’s pentachrome staining and image quantification

Movat's pentachrome stain was utilised to determine the percentage area occupied by elastic fibres (appearing as black to blue/black) and total collagen (appearing as yellow to red) within the submucosa of the bronchial biopsy tissue sections. Additionally, the stain allowed visualisation of muscle (in red), mucin (in bright blue), and fibrin (in bright red) for histological assessment. Computerised image analysis software (Image J) was employed to measure the percentage of elastin and collagen as a fraction of the total submucosal area. Initially, the areas of unrecognizable tissue and blood vessels were excluded from the region of interest, which was defined using the software. The total area of interest was then calculated by the software. To accurately quantify the area of positive staining, colour thresholding techniques were applied to distinguish positively stained regions from background tissue based on predefined RGB hue threshold values, as previously described (1,2). This approach enabled automated identification of stained areas and allowed the total area of positive staining to be quantified as a proportion of the total submucosal area.

## Supplementary Figure Legends

### Figure S1. Distribution of elastic fibres in the airways.

(A) main bronchus. (B) bronchial gland. (C) segmental bronchus. (D) terminal/respiratory bronchiole. (E) alveoli. (F) Fluorescent alveolar macrophages in active smokers. In the more proximal airways such as the trachea and main bronchi, the elastic fibres lie longitudinally within the airway wall although this organisation gets disrupted at the carinae and around the openings of the bronchial mucous glands (3). This contrasts with the more distal airways represented by the interlacing lattice-like pattern made of 2- to 5-μm thick fibres at the bronchiolar level and the distinct helicoidal organization of the axial connective tissue network around the respiratory bronchioles and alveoli (3). Hyperfluorescent alveolar macrophages are typically seen in current smokers (4).

### Figure S2. pCLE image of an alveolar opening in a patient with COPD.

The long axis alveolar diameter (LA) (green) and the perpendicular short axis (SA) (blue). The mean values of alveolar dimensions over several respiratory cycles were obtained as these were measured during spontaneous breathing as previously described (5,6)

.

### Figure S3. Workflow for derivation of the elastin linearity score (ELS)

Probe-based confocal laser endomicroscopy (pCLE) video sequences were acquired during bronchoscopy. Individual frames were processed using a bank of Gabor and Gaussian filters to detect elastin fibres, estimate their dominant orientation, and enhance linear features. Images were subsequently thresholded and processed with morphological operators to generate single-pixel-width fibre representations. The standard deviation of fibre orientation (θ) was calculated for each frame, and the mean across all frames per subject was derived to yield a single measure of angular dispersion, the elastin linearity score (ELS). Higher ELS values indicate greater elastin fibre disorganisation.

Abbreviations: CNN, convolutional neural network; SD, standard deviation; θ, fibre orientation angle.

### Figure S4: Example of Imague used to determine ELS

(Top) Example of detection of the dominant direction at every point in the image. Left - Original pCLE image, Right - Image coloured to show the detected dominant direction (red/yellow indicates fibres orientated from bottom left to top right, blue/green indicates fibres orientated from top left to bottom right, with different shades indicated interpolation between these two extremes).

(Bottom) Example of enhancement of linear features. Left - Original image, Right - Enhanced features (red/black indicates a high response corresponding to a strongly linear features, whilst blue/green indicates a lower response suggesting that no linear features were present)

## Supplementary Tables

|  | Controls with preserved lung function | COPD | P value |
| --- | --- | --- | --- |
| Subjects, n | 12 | 9 | - |
| Age, years | 49 (8.7) | 61 (3.9) | **0.0044** |
| Sex (M/F), n (% Males) | 9/3 (75%) | 8/1 (89%) | 0.4225 |
| Smoking history (Never/Ex/Current)) | 8/0/4 | 0/4/5 | **0.0026** |
| Smoking pack year | 0 (17.4) | 42 (19.7) | **0.0011** |
| FEV1% of predicted | 102.8 (15.1) | 82.1 (13.3) | 0.1005 |
| FEV1/FVC (%) | 78.4 (4.2) | 57.9 (7.4) | **0.0251** |
| MEF25-75% | 85.7 (23.6) | 30.1 (13.6) | 0.0529 |
| TLCO % of predicted | 88.5 (12.0) | 83.0 (16.8) | 0.8578 |
| E/I MLD median | 0.76 (0.06) | 0.86 (0.04) | **<0.0001** |
| HRCT %LAA-950 | 2.48 (1.68) | (9.35 (6.45) | **<0.0001** |

### Table S1. Demographics of the biopsy cohort (a sub-cohort of the pCLE cohort).

The 16 controls with preserved lung function included 8 controls never smokers and 4 control current smokers). Statistical testing performed using chi-squared for categorical variables (Sex: male or female) and Mann-Whitney U test for continuous variables (all other variables). Data are presented as median (Standard deviation) unless otherwise indicated. Significant values in bold.

|  | Controls | COPD | p value |
| --- | --- | --- | --- |
| Subjects, n | 61 | 54 | - |
| Age, years | 59 (11) | 67 (8) | **< 0.0001** |
| Sex (M/F), n (% males) | 34/27 (55.7%) | 40/14 (74.1%) | **0.0240** |
| Smoking history (Never/Ex/Current) | 29/22/10 | 0/43/11 | **< 0.0001** |
| Smoking pack year | 10 (17.2) | 42 (22.7) | **< 0.0001** |
| FEV1% of predicted | 104.5 +/- 14 | 74.2 +/- 16.7 | **< 0.0001** |
| FEV1/FVC (%) | 78.8 +/- 4.7 | 56.5 +/- 9.4 | **< 0.0001** |
| MEF25-75% | 103.3 +/- 28.5 | 35.8 +/- 15.2 | **< 0.0001** |
| TLCO % of predicted | 90.0 (13.3) | 81.0 (14/5) | **0.0009** |
| E/I MLD median | 0.79 (0.06) | 0.86 (0.05) | **<0.0001** |
| HRCT %LAA-950 | 4.2 (4.1) | 9.1 (7.7) | **<0.0001** |

### Table S2. Demographics of the Neoepitope cohort.

The Neotepitope cohort was comprised of the pCLE cohort (above in Table 1) and the previously reported MCIA II cohort (above in Table 2). Statistical testing performed using chi-squared for categorical variables (Sex: male or female) and Mann-Whitney U test for continuous variables (all other variables). Data are presented as median and (Standard deviation) unless otherwise indicated.

| **Biomarker** | **Specifications** | **Measure** |
| --- | --- | --- |
| PRO-C3 | N-terminal pro-peptide of type III collagen | Formation of interstitial matrix, fibroblast activity (7) |
| PRO-C4 | 7S domain of type IV collagen | Turnover of basement membrane (8) |
| PRO-C6 | C5 domain of type VI collagen α3 chain | Formation of interstitial matrix, fibroblast activity (9) |
| C1M | Degradation of type I collagen by MMP-2/9/13 | Break-down of interstitial matrix (10) |
| C3M | Degradation of type III collagen by MMP-9 | Break-down of interstitial matrix (11) |
| C4M | Degradation of type IV collagen by MMP-2/9/12 | Break-down of basement membrane (12) |
| C6M | Degradation of type VI collagen by MMP-2 | Break-down of interstitial matrix (13) |
| ELP-3 | Degradation of elastin by proteinase 3 | Neutrophil-related break-down of interstitial matrix (14) |
| EL-CG | Degradation of elastin by cathepsin G | Neutrophil-related break-down of interstitial matrix (15) |
| EL-NE | Degradation of elastin by neutrophil elastase | Neutrophil-related break-down of interstitial matrix (16) |

### Table S3. Elastin and collagen neoepitope biomarkers.

|  | Submucosal tissue collagen percentage | Submucosal tissue elastin percentage |
| --- | --- | --- |
| FEV1% | **-0.434*** | -0.170 |
| FEV1/FVC | **-0.629**** | -0.208 |
| MEF25%-75% | **-0.701**** | -0.198 |
| TLCO% | 0.090 | 0.085 |
| E/I MLD | **0.543*** | -0.194 |
| %LAA-950 | **0.549**** | -0.026 |
| SA | 0.358 | 0.168 |
| LA | **0.611**** | -0.140 |
| AOA | **0.507*** | 0.079 |
| ELS | **0.665**** | 0.018 |

### **Table S4. Spearman's correlation analysis between the submucosal tissue collagen and elastin and lung function, CT and pCLE measures.**

Staining performed on biopsy cohort, 12 controls with preserved lung function (8 control never smokers and 4 control current smokers) and 9 COPD subjects (4 current smokers and 5 ex-smokers). Numbers in the boxes are the r value of statistically significant correlations *= p<0.05, **= p<0.01.

|  | Adjusted OR [95% CI] | P-value |
| --- | --- | --- |
| EL-NE (ng/mL) | 0.94[0.60_1.45] | 0.7639 |
| C3M (ng/mL) | 1.11[0.97_1.26] | 0.1267 |
| PRO-C4 (ng/mL) | 1.00[1.00_1.01] | 0.0388 |
| PRO-C3 (ng/mL) | 1.06[0.87_1.30] | 0.5635 |
| EL-CG (ng/mL) | 1.21[1.01_1.46] | 0.0396 |
| C1M (ng/mL) | 1.03[1.01_1.05] | 0.0098 |
| C4M (ng/mL) | 1.05[0.99_1.11] | 0.1125 |
| C6M (ng/mL) | 1.07[1.01_1.13] | 0.0202 |
| PRO-C6 (ng/mL) | 1.03[0.86_1.24] | 0.7337 |
| ELP-3 (ng/mL) | 1.01[1.00_1.02] | 0.0945 |

### Table S5. logistic model fitted to regress Case/ Control status on neoepitope, adjusting for smoking group, age, and sex.

Neoepitopes were measured across 115 individuals (54 with COPD; 61 Healthy volunteers), 10 of which were current smokers. Histograms were drawn to display neoepitope data, and for the purposes of parametric analysis, three outliers were removed (EL-NE=10.073 ng/ml; PRO-C3=32.79 ng/ml; C1M=218.8 ng/ml). Smoking group was analysed instead as a quantitative (dose) parameter, with values 1,2, and 3.

|  | LA | SA | AOA |
| --- | --- | --- | --- |
| FEV1% | -0.359^*^ | -0.464^**^ | -0.465^**^ |
| FEV1/FVC | -0.706^**^ | -0.637^**^ | -0.721^**^ |
| MEF25%-75% | -0.656^**^ | -0.623^**^ | -0.724^**^ |
| TLCO% | -0.382^*^ | -0.345 | -0.345 |
| E/I MLD | 0.697^**^ | 0.612^**^ | 0.677^**^ |
| %LAA-950 | 0.495** | 0.564** | 0.489** |

### **Table S6. Spearman's correlation analysis between alveolar dimensions, lung function and CT measures in the pCLE cohort.**

Numbers in the boxes are the r value of statistically significant correlations *= p<0.05, **= p<0.01.

**References:**

1.	Clift CL, Su YR, Bichell D, Jensen Smith HC, Bethard JR, Norris-Caneda K, et al. Collagen fiber regulation in human pediatric aortic valve development and disease. Sci Rep. 2021 Dec 1;11(1). doi:10.1038/s41598-021-89164-w PubMed PMID: 33963260.

2.	Collins TJ. ImageJ for Microscopy. Biotechniques. 2007 Jul;43(sup1). doi:10.2144/000112517 PubMed PMID: 17936939.

3.	Thiberville L, Moreno-Swirc S, Vercauteren T, Peltier E, Cavé C, Heckly GB. In Vivo imaging of the bronchial wall microstructure using fibered confocal fluorescence microscopy. Am J Respir Crit Care Med. 2007 Jan 1;175(1):22–31. doi:10.1164/rccm.200605-684OC PubMed PMID: 17023733.

4.	Newton RC, Kemp S V., Yang GZ, Elson DS, Darzi A, Shah PL. Imaging parenchymal lung diseases with confocal endomicroscopy. Respir Med. 2012 Jan;106(1):127–37. doi:10.1016/j.rmed.2011.09.009 PubMed PMID: 22000588.

5.	Cosío BG, Shafiek H, Fiorentino F, Gómez C, López M, Rios A, et al. Structure-function relationship in COPD revisited: An in vivo microscopy view. Thorax. 2014;69(8):724–30. doi:10.1136/thoraxjnl-2013-204479 PubMed PMID: 24477204.

6.	Thiberville L, Saiaiin M, Lachkar S, Dominique S, Moreno-Swirc S, Vever-Bizet C, et al. Human in vivo fluorescence microimaging of the alveolar ducts and sacs during bronchoscopy. European Respiratory Journal. 2009 May;33(5):974–85. doi:10.1183/09031936.00083708 PubMed PMID: 19213792.

7.	Nielsen MJ, Nedergaard AF, Sun S, Veidal SS, Larsen L, Zheng Q, et al. The neo-epitope specific PRO-C3 ELISA measures true formation of type III collagen associated with liver and muscle parameters. Am J Transl Res [Internet]. 2013. Available from: www.ajtr.org

8.	Leeming DJ, Nielsen MJ, Dai Y, Veidal SS, Vassiliadis E, Zhang C, et al. Enzyme-linked immunosorbent serum assay specific for the 7S domain of collagen type IV (P4NP 7S): A marker related to the extracellular matrix remodeling during liver fibrogenesis. Hepatology Research. 2012 May;42(5):482–93. doi:10.1111/j.1872-034X.2011.00946.x PubMed PMID: 22221767.

9.	Sun S, Henriksen K, Karsdal MA, Byrjalsen I, Rittweger J, Armbrecht G, et al. Collagen type III and VI turnover in response to long-term immobilization. PLoS One. 2015 Dec 1;10(12). doi:10.1371/journal.pone.0144525 PubMed PMID: 26641456.

10.	Leeming DJ, He Y, Veidal SS, Nguyen QHT, Larsen D V., Koizumi M, et al. A novel marker for assessment of liver matrix remodeling: An enzyme-linked immunosorbent assay (ELISA) detecting a MMP generated type I collagen neo-epitope (C1M). Biomarkers. 2011 Nov;16(7):616–28. doi:10.3109/1354750X.2011.620628 PubMed PMID: 21988680.

11.	Barascuk N, Veidal SS, Larsen L, Larsen D V., Larsen MR, Wang J, et al. A novel assay for extracellular matrix remodeling associated with liver fibrosis: An enzyme-linked immunosorbent assay (ELISA) for a MMP-9 proteolytically revealed neo-epitope of type III collagen. Clin Biochem. 2010 Jul;43(10–11):899–904. doi:10.1016/j.clinbiochem.2010.03.012 PubMed PMID: 20380828.

12.	Sand JM, Larsen L, Hogaboam C, Martinez F, Han ML, Larsen MR, et al. MMP mediated degradation of type IV collagen alpha 1 and alpha 3 chains reflects basement membrane remodeling in experimental and clinical fibrosis - Validation of two novel biomarker assays. PLoS One. 2013 Dec 23;8(12). doi:10.1371/journal.pone.0084934 PubMed PMID: 24376856.

13.	Veidal SS, Karsdal MA, Vassiliadis E, Nawrocki A, Larsen MR, Nguyen QHT, et al. MMP mediated degradation of type VI collagen is highly associated with liver Fibrosis - Identification and validation of a novel biochemical marker assay. PLoS One. 2011 Sep 14;6(9). doi:10.1371/journal.pone.0024753 PubMed PMID: 21935455.

14.	Gudmann NS, Manon-Jensen T, Sand JMB, Diefenbach C, Sun S, Danielsen A, et al. Lung tissue destruction by proteinase 3 and cathepsin G mediated elastin degradation is elevated in chronic obstructive pulmonary disease. Biochem Biophys Res Commun. 2018 Sep 10;503(3):1284–90. doi:10.1016/j.bbrc.2018.07.038 PubMed PMID: 30017196.

15.	Gudmann NS, Manon-Jensen T, Sand JMB, Diefenbach C, Sun S, Danielsen A, et al. Lung tissue destruction by proteinase 3 and cathepsin G mediated elastin degradation is elevated in chronic obstructive pulmonary disease. Biochem Biophys Res Commun. 2018 Sep 10;503(3):1284–90. doi:10.1016/j.bbrc.2018.07.038 PubMed PMID: 30017196.

16.	Kristensen JH, Karsdal MA, Sand JMB, Willumsen N, Diefenbach C, Svensson B, et al. Serological assessment of neutrophil elastase activity on elastin during lung ECM remodeling. BMC Pulm Med. 2015 May 3;15(1). doi:10.1186/s12890-015-0048-5 PubMed PMID: 25935650.
